# The effect of selection on allele frequencies and diversity in wheat (*Triticum aestivum*)

**DOI:** 10.1007/s00122-026-05367-7

**Published:** 2026-10-01

**Authors:** Mirai Inaoka, Carlos Ignacio, Nelly Arguello-Blanco, Lenin Rodrigues Betancourth, Clay Sneller

**Affiliations:** 1 The Ohio State University, 1680 Madison Av, Wooster, OH 44691 USA; 2 Grand Farm, University of Georgia, Perry, GA 31047 USA

## Abstract

**Key message:**

Advancing wheat lines through stages of testing resulted in large genome changes. Consistent changes across cohorts and selection stages were noted though most changes appeared specific. Diversity was lost within a cohort, primarily at the last stage of advancement, though diversity across cohorts was not greatly impacted. The results can be used to develop informed crossing strategies.

**Abstract:**

Effective selection increases the frequency of favorable alleles but can reduce genetic variation. The extent of allele frequency changes (AFC) that occur as wheat lines are advanced through stages of testing is not well documented. Our objectives were to determine the magnitude of AFC in a wheat breeding program, determine the consistency of AFC, and the effect of selection on population structure and diversity. We calculated the AFC for 1,173 markers on 4,678 wheat lines from ten cohorts that went through four stages of selection, primarily for yield. We determined the AFC for 48,093 marker/cohort/selection stage combinations and determined that 23.5% of these AFC’s were greater than expected by drift and were attributed to selection. Most large AFC occurred in the final stage of selection. Eighty-nine markers had AFCs that showed signs of consistent unidirectional selection over cohorts and stages of selection, while 68.3% of the AFCs attributed to selection were cohort/selection-stage and directional specific. Within a cohort an average of 24.1% of makers had become fixed in stage−4 of testing, though few markers become fixed in more than one cohort. Each cohort experienced somewhat unique genome changes, likely due to selection in a unique array of environments. The genetic distance between cohorts of lines in stage−4 testing was similar to that among stage−1 lines. Crossing among the best lines from multiple cohorts would mitigate the loss of diversity within a single cohort and likely recombine favorable alleles selected across a wide array of environments.

**Supplementary Information:**

The online version contains supplementary material available at https://doi.org/10.1007/s00122-026-05367-7.

## Introduction

The wheat breeding program at The Ohio State University (OSU) began around 1860 with a focus on soft red winter wheat (Klippert 1857; National Rural and Family Magazine 1899). These early breeding efforts were limited to evaluating germplasm for adaptation to Ohio, followed by a shift to the selection of superior individuals from the older heterogeneous cultivars. Hybridization and selection of superior progeny was initiated about the time of World War I. The program now utilizes traditional and molecular breeding techniques including genomic selection (Borrenpohl et al. 2020; Meuwissen et al. 2001). Our current germplasm is a result of > 110 years of crossing, recombination, introduction of new germplasm, and extensive selection.

Intensive selection for elite trait levels through phenotypic or genomic selection can lead to a loss of genetic diversity that can limit future gains from selection (Arguello-Blanco and Sneller 2023, Jannink, 2010, Voss-Fels et al., 2015). Understanding changes in the wheat genome that occur during selection within a breeding program will enable plant breeders to set an optimal selection intensity that prioritizes the gain from selection while still maintaining overall diversity of the genome (Jannink 2010). Establishing an optimal contribution selection (OCS) is one method to minimize loss of genetic diversity and maximize the long-term response to selection, as it helps to increase selection gains in an inbreeding population by optimizing the genetic contribution of each individual in the next generation (Cowling et al. 2017; Gorjanc et al. 2018; Woolliams et al. 2015).

There have been some studies seeking to understand the effect of selection on the wheat genome. Ayalew et al. (2020) identified selection signatures among 185 commercially released wheat cultivars by calculating Wright’s fixation indices estimated for different subpopulations used in the study. They discovered that breeding for bread wheat in the Great Plains of the USA did not cause major reductions in genetic diversity, and most selection signatures were associated with disease resistance genes. On the other hand, Mondaini et al. (2022) discovered that 9.8% of loci exhibited strong evidence of selection over 38 years of breeding in CIMMYT’s elite bread wheat lines. They identified these loci by assessing the pattern of variance in allele frequencies. Arguello-Blanco and Sneller (2023) report significant changes in the wheat genome following five cycles of genomic selection, such that the individuals in cycle five appeared unrelated to the training population based on Fst values.

These studies do not investigate changes within a single cohort as its members are advanced through successive stages of testing. Studies such as Ayalew et al. (2020) and Mondaini et al. (2022) also do not restrict their analyses to one elite breeding program, making it difficult to identify signatures of selection within a cohort.

## Materials and methods

### Populations

The wheat population used in this study consists of 4673 soft red winter wheat lines from 10 cohorts in the OSU wheat breeding program (Table 1). A cohort is defined as a set of lines that were created together and first evaluated together in the same year. For example, the OH16 cohort consists of all lines that were selected from head rows in 2016 and entered stage−1 yield testing together in the 2016–2017 season.

**Table 1:** Summary of number of genotyped wheat lines in each cohort and stage of testing, and the number of locations (locs) and replications (reps) in each stage of testing

	Stage 1			Stage 2			Stage 3			Stage 4		
Cohort	n	# Locs	# Reps	n	# Locs	# Reps	n	# Locs	# Reps	n	# Locs	# Reps
OH13	**–**	**–**	**–**	**–**	**–**	**–**	37	3	3	8	6	4
OH14	**–**	**–**	**–**	234	3	1	38	3	2	6	6	3
OH15	**–**	**–**	**–**	251	3	1	72	3	2	15	3	3.3
OH16	466	1	1	248	3	1	44	3	2	15	6	3.3
OH17	651	1	1	222	2*	1	52	3	2	11	6	4
OH18^G^	786	1	1	240	2*	1	56	3	2	8	6	3.3
OH19^G^	614	1	1	256	2*	1	81	4	2	11	6	3.3
OH20^G^	632	1	1	129	2*	1	34	4	2	10	7	3.2
OH21^G^	495	1	1	124	2*	1	58	4	2	**–**	**–**	**–**
OH22^G^	498	1	1	147	2*	1.3	**–**	**–**	**–**	**–**	**–**	**–**

*Sparse testing: Each line was grown in 2 out of 3 locations. ^G^ Genomic selection was used to advance lines from stages 1, 2 and 3

### Phenotypic data

The program phenotypes lines through four stages of field trials with selection for yield, test weight, resistance to Fusarium Head Blight (FHB, *Fusarium graminearum*), and agronomic traits conducted at each stage. Resistance to FHB was assessed in a disease evaluation nursery at Wooster, Ohio, generally with one rep for stage−1 trials, two reps for stage−2 trials, and 2–3 reps for stage−3 and − 4 trials. Yield, test weight and agronomic traits data were collected as follows. The stage−1 trial was conducted at one location (Wooster, Ohio) with one replication (Table 1). Lines in the stage−2 trial were grown at three Ohio locations for cohorts OH13 to OH16 with one replication. Sparse testing was implemented for the stage−2 trials for cohorts OH17 to OH22 with each line grown in two of three locations with genomic selection was used to estimate their value at the third location (Table 1). The stage−3 trial was conducted at three Ohio locations for cohorts OH13 to OH18 and at four locations for cohorts OH19 to OH21. Stage−3 trials had two to three replications. The stage−4 trial was conducted at six to seven Ohio locations with three to four replications. Genomic selection was used beginning in 2019 with the OH18 cohort to complement selection based on phenotypes to advance lines from stages 1 and 3.

Selection for FHB resistance is primarily based on a visual rating for FHB index, which is the percentage of all spikelets in a plot that display symptoms of infection. The data are collected from plots in a misted and inoculated FHB nursery as described by Sneller et al. (2010). The nursery consists of single 1 M row plots that are inoculated with spores and misted as described by Hoffstetter et al. (2016). Index is visually estimated at three areas per plot. Grain yield and test weight data are measured by harvesting plots with a Wintersteiger Classic combine equipped with a HarvestMaster weigh system. Grain yield per plot was converted to kilograms per hectare at 13% moisture and test weight was converted to kilograms per hectoliter at 13% moisture. Criteria for selecting lines to advance to the next stage of trials were somewhat fluid with compromises made. In general, in order to be advanced, lines need to 1) yield no more than 100 kg/ha lower than the checks, 2) have a test weight no more than 5% below average, 3) have an FHB index no greater than that of the moderate resistance check, 4) have a heading date no more than two days later than the maturity check, and 5) measure no greater than six cm taller than the check. For example, a line with exceptional yield, but with below average test weight could be advanced, while a line with exceptional FHB resistance could also be advanced despite having a yield somewhat below the checks. The percentage of lines advanced between stages is shown in Table 2 for each cohort. It should be noted that for some of these traits, nearly all lines meet the “acceptable” criteria. Currently, nearly 80% of all lines meet the “acceptable” criteria for FHB index, test weight, heading date, and height, thus shifting the focus of selection within the breeding program primarily for superior yield.

**Table 2:** Percentage of wheat lines from each cohort that were advanced from one stage of testing to the next stage

	Selection Stage					
Cohort	1 to 2*	1 to 3	1 to 4	2 to 3	2 to 4	3 to 4
OH13	**–**	**–**	**–**	**–**	**–**	21.6
OH14	**–**	**–**	**–**	16.0	2.5	15.8
OH15	**–**	**–**	**–**	28.7	6.0	20.8
OH16	53.2	9.4	3.2	17.7	6.0	34.1
OH17	34.1	8.0	1.7	23.4	5.0	21.2
OH18	30.5	7.1	1.0	23.3	3.3	14.3
OH19	41.7	13.2	1.8	31.6	4.3	13.6
OH20	20.4	5.4	1.6	26.4	7.8	29.4
OH21	25.1	11.7	–	46.8	–	–
OH22	29.5	–	–	–	–	–
Average	33.5	9.1	1.9	26.7	5.0	21.3

*“1 to 2” indicates advancing lines from stage−1 trial to a stage−2 trial

### Genotyping and marker data

DNA from 2-week-old plants was extracted in 96-well plates following the DNeasy® 96 Plant Kit (Qiagen 2022) protocol. The individuals were genotyped at the wheat genetics laboratory at Kansas State University. Marker data for the lines used in this study were obtained according to the genotyping-by-sequencing (GBS) protocol (Poland et al. 2012). The resulting single nucleotide polymorphisms (SNPs) were called using the TASSEL-GBS pipeline (version 4.3.5) (Glaubitz et al. 2014) and filtered using the pipeline outlined in Ward et al. (2019). Variant calling discovered more than 694k SNPs. SNPs with minimum allele frequency less than 5% and heterozygous calls greater than 25% were removed, leaving a total of 3,452 SNPs. Since we worked with inbred lines, very little heterozygosity was expected. The remaining SNPs had less than 20% of missing data that were then imputed using Beagle version 5.5 (Browning et al. 2018) with a genetic map of 906 recombinant inbred wheat lines derived from Synthetic × Opata cross (Gutierrez-Gonzales et al. 2019). Beagle version 5.5 performs genotype imputation based on identity by descent (IBD) (Browning et al. 2018). The marker dataset was converted from HapMap format to numeric using CropGalaxy (The Galaxy Community 2024). Genotypes in the marker data were encoded as 0, 1, and 2 for being homozygous for the major allele, heterozygous, or homozygous for the alternate allele, respectively. Finally, SNPs in high linkage disequilibrium (${r}^{2}<$ 0.4) were pruned using PLINK 1.9 (Chang et al. 2015) with a 250kb-SNP sliding window and advanced 10 SNPs at each step, resulting in a final SNP count of 1,173. The cutoff (${r}^{2}<$ 0.4) was used so as not to lose all markers with the filtering.

### Allele frequencies

The frequency of the most common allele, termed the major allele, across all 4,673 lines was determined for each marker. Marker genotype data were encoded as 0, 1, and 2 for being homozygous for the minor allele, heterozygous, or homozygous for the major allele, respectively. The frequency of this major allele (MAF) was calculated for each set of lines, from each cohort, in each stage of trialing, which created 33 subsets of lines (Table 1). For example, we created two sets of lines for the OH13 cohort: those tested in stage−3 and those tested in stage−4. In this example, the lines in the stage−3 subset are also in the stage−4 subset. It is important to note that the major allele was determined across all 4,673 lines, so the frequency of this allele could be less than 0.50 in some subsets. The MAF of a marker in each of the 33 subsets was calculated by taking the column mean and dividing by two. This calculation was repeated for all 1,173 markers in all 33 subsets using scripts written in R version 4.4.2 (R Core Team 2024) by the authors.

Our data presented six selection scenarios where allele frequency change (AFC) could be calculated: selection of stage−1 lines advanced to stage−2 (1→2), stage−2 to stage−3 (2→3), 3→4, 1→3, 1→4, and 2→4 (Table 2). In total, there were 41 combinations of cohort and selection scenarios, as some cohort/selection scenarios were not possible (Table 2). At the time of the analysis, selections from the OH22 cohort stage−2 trial had not been made, so no 2→3 or 3→4 analyses were possible. Also, not all OH13, OH14, and OH15 lines grown in the stage−1 trial were genotyped, so the MAF for lines from these cohorts in stage−1 could not be calculated.

The observed AFC for the major allele from the advancing of one marker in one of the 41 cohort/selection scenarios from advancement from the *i*^th^ stage of testing to the *i*^th+1^ stage of testing was calculated as

1 $$AFC= {f}_{i}-{f}_{i+1}$$

where *f*_*i*_ is the frequency of the major allele in the i^th^ stage and *f*_*i*+*1*_ is the frequency of the same allele in the i^th^+1 stage. This calculation was repeated for all 1,173 markers and 41 cohort/selection scenario combinations, providing 48,093 estimates of AFC.

### Genetic drift simulation

**Fig. 1:**
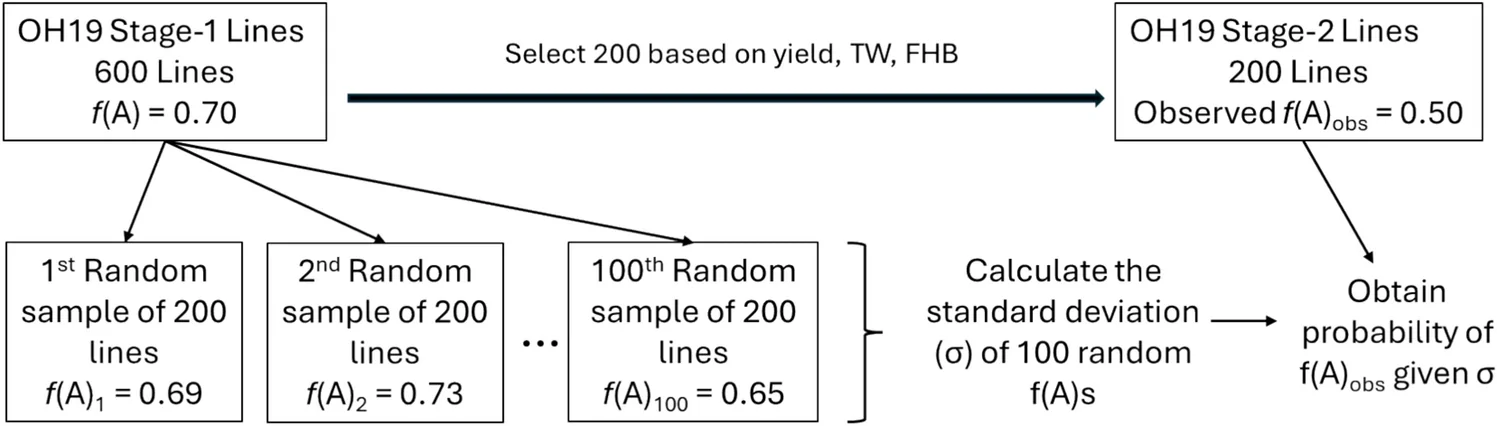
Diagram of analytical pipeline used to simulate drift and the probability of an allele frequency change for one marker being attributed to drift within the OH19 cohort as lines were advanced from stage−1 to stage−2

An observed AFC could be due to selection or genetic drift, defined as allele frequencies changing purely by random chance (Hartl and Clark 1997). We simulated the probability distribution of AFC that could arise by random chance when advancing x of m lines where x < m. for the major allele for all genetic markers, cohorts, and selection scenarios. The simulation was conducted using an analyses pipeline in R version 4.4.2 (R Core Team 2024). The simulation was run for each of the 48,093 marker/cohort/selection scenario combinations. One analysis was conducted by randomly sampling *x* lines from the i^th^ testing stage where *x* was the number of lines from a cohort that were advanced from the i^*th*^ stage to the *i*^*th*^+1 stage of testing. Then, the MAF was calculated within the random sample of *x* lines. This sampling and calculation procedure was repeated 100 times for each of the 48,093 instances to generate 100 MAF values that occurred by random chance. A randomization seed was set to ensure reproducibility of the results. For example, the OH19 cohort had 600 lines in the stage−1 trial, of which 200 were selected to advance to the stage−2 trial (*x* = 240) (Fig. 1). We randomly selected 200 of the 600 lines, calculated the MAF value, and repeated this process 100 times. The standard deviation of these 100 MAF values, σ, was calculated. The probability of the observed AFC occurring by chance alone was calculated by the following equation:

2 $$p=2 \times (1-\phi \frac{\left|AFC\right|}{\sigma })$$

where *p* represents the probability that the observed AFC would occur by random chance and ϕ represents the cumulative distribution function of the standard normal distribution. AFC with a p <0.05 were deemed to be attributed to selection and designated as AFCsel. The entire analysis of the 48,093 marker/selection scenario combinations required 4,809,300 estimates of random AFC.

### Assessment of consistency of AFCs

We assessed the consistency of changes in AFC for each of the 1,173 markers across all of the applicable 41 cohort/selection scenarios combinations by calculating several consistency parameters and establishing cutoff values for each. The parameters were as follows:

1. %AFCsel: percentage of ~41 scenarios where the probability of the observed AFC occurring by drift was ≤ 0.05. For example, if a marker had a probability of drift ≤ 0.05 in 25 of 41 cohort/selection scenarios, then the percentage of AFCsel scenarios was 60.1%.
2. #PosAFC: Number of scenarios with AFC > 0.05
3. #NegAFC: Number of scenarios with AFC < − 0.05

We declared that a marker showed consistent change over selection scenarios if the %AFCsel was ≥ 35%, which was > 1.5 standard deviations above the mean %AFCsel percentage of 23.85%. An AFCsel for any one marker could involve an increase or decrease in the MAF. The consistency of the direction of AFC was assessed by calculating the ratio of times the AFC in one direction (larger of #PosAFC or #NegAFC) and the times it changed in the other direction (Smaller of #PosAFC or #NegAFC). For example, if #PosAFC was 15 and #NegAFC was 3 then the ± ratio was determined to be 15/3=5. Markers with ± ratios of ≥ 3 were and with %AFCsel > 35% were deemed to be consistent.

### Determination of allele fixation

Markers were declared fixed when the post-selection frequency (*f*_*i*+*1*_) of one allele was greater than 0.99, and the pre-selection frequency (*f*_*i*_) of the same allele was less than 0.96. The values allow for some ambiguities in the GBS genotyping.

### Statistical model

The change in phenotypic values within a cohort as the cohort progressed through stages of selection were assessed. The best linear unbiased estimate (BLUE) was obtained for each line within each cohort using only trait data from lines within that cohort. The following model was used to obtain the BLUEs:

3 $${y}_{ijkl}= {\mu + g}_{i}+ {{y}_{j}+ l}_{k}+ {r(ly)}_{ijkl}+ {gy}_{ij}+ {gl}_{ik}+ {gly}_{ijk}+ {\varepsilon}_{ijkl}$$

where ${g}_{i}$ was the fixed effect of the *i*^th^ line, ${y}_{j}$ was the random effect of the *j*^th^ year with ${y}_{kj}\sim N(0, {\sigma}_{y}^{2}$), ${l}_{k}$ was the random effect of the* k*^*th*^ location with ${l}_{k}\sim N(0, {\sigma}_{l}^{2}$), ${r(ly)}_{ijkl}$ was the random effect of the *l*^*th*^ rep nested in year and location with ${r(ly)}_{ijkl}\sim N(0, {\sigma}_{r(yl)}^{2}$), ${gy}_{ij}$ was the random effect of the interaction of the *i*^th^ genotype and *j*^th^ year with ${gy}_{ij}\sim N(0, {\sigma}_{gy}^{2}$), ${gl}_{ik}$ was the random effect of the interaction of the *i*^th^ genotype and the *k*^th^ location with ${gl}_{ik}\sim N(0, {\sigma}_{gl}^{2}$), ${gly}_{ijk}$ was the interaction of the *i*^th^ genotype with the* j*^*th*^ year and *k*^th^ location with ${gly}_{ijk}\sim N(0, {\sigma}_{gly}^{2}$) and ${\varepsilon}_{ijkl}$ was the error with ${error}_{ijkl}\sim N(0, {\sigma }^{2}$).

The BLUEs for each line were then regressed on to its last stage of testing (1, 2, 3, or 4). The slope from the regression represents the estimate of the trait change as lines progress from one stage of testing to the next.

### Genome-wide association study

A genome-wide association study (GWAS) was conducted using data from all 1,173 SNPs and data for grain yield, test weight, FHB index, maturity, height, and appearance score (scored as 1=very good, 5= very poor). A multiple locus mixed linear model was run on the BLUEs obtained from running the statistical model shown in Eq. 3 where all data from all lines and cohorts were included in one analysis. The software GAPIT (Lipka et al. 2012) was used to conduct the GWAS for each trait, with three principal components included in the model as fixed effects of population structure.

### Population structure

The degree of differentiation between cohorts in different stages of selection was estimated using the fixation index (F_ST_, Weir and Cockerham 1984). The degree of fixation among the lines in different cohorts and stages of testing was computed using the *hierfstat* package version 0.5–11 in R (Goudet 2005; R Core Team 2024) that uses the following formula to compute for F_ST_:

4 $${F}_{ST}\left(a,b\right)= -\mathrm{l}\mathrm{n}(\frac{{\sum}_{k=1}^{v}{\sum}_{j=1}^{m\left(k\right)}{p}_{ajk}{p}_{bjk}}{\sqrt{\sum_{k=1}^{v}{\sum}_{j=1}^{m\left(k\right)}{\left({p}_{ajk}\right)}^{2}\sqrt{{\sum}_{k=1}^{v}{\sum}_{j=1}^{m\left(k\right)}{\left({p}_{bjk}\right)}^{2}}}})$$

where *a* and *b* represent the two populations, ${p}_{ajk}$ represents the frequency of the *j*^th^ allele of the *k*^th^ marker in population *a*, and ${p}_{bjk}$ represents the frequency of the *j*^th^ allele of the *k*^th^ marker in population *b*. Each line was placed into one of 33 groups according to their cohort and stage of testing. For example, OH16 lines that were only tested in stage−1 were placed in the OH16-1 group, OH16 lines that were tested in stages 1 and 2 were placed in the OH16-2 group, etc. Ward’s hierarchical clustering method (Ward, 1963) was performed on the F_ST_ matrix in R (R Core Team 2024).

#### Linkage disequilibrium

A matrix of linkage disequilibrium (LD) between markers was calculated between all pairs of 89 SNPs with consistent AFC using the following formula:

5 $${r}^{2}= \frac{{D}^{2}}{pA\left(1-pA\right)pB(1-pB)}$$

6 $$D= {p}_{A}- {p}_{A}{p}_{B}$$

where D is estimated between two loci (A, B), each with two alleles (A1 and A2, B1 and B2) producing *A1B1*, *A1B2*, *A2B1*, *A2B2* haplotypes with frequencies ×11, ×12, ×21, and ×22, respectively. Allele frequencies are equal to *ppAA* = ×*11* + ×*12* and *ppBB* = ×*11* + ×*21*. The ${r}^{2}$ value was estimated using the LD function in TASSEL version 5.0 (Lipka et al. 2012).

#### Genetic distance

We quantified genetic distance between pairs of individuals using an identity-by-state (IBS) metric, which accounts for the number of alleles shared between two lines at each locus. For two individuals i and j, let ${g}_{i{\ell}},{ g}_{j{\ell}}\in \{\mathrm{0,1},2\}$ denote the diploid genotypes at locus ℓ(0= homozygous reference, 1= heterozygous, 2= homozygous alternate). For each locus, we assign an IBS identity score:

$${IBS}_{ij}^{({\ell})}=\left\{\begin{array}{c}1\\ 0.5\\ 0\end{array}\right. \begin{array}{c}if {g}_{i{\ell}}={g}_{j{\ell}}(same genotype)\\ if \,one \,genotype \,is \,heterozygous\, and\, the\, other\, homozygous\,\\ if\, genotypes\, are\, opposite\, homozygotes\, (0 vs. 2)\end{array}$$

Across all loci with non-missing genotypes in both individuals, the pairwise IBS identity is given by

$${IBS}_{ij}\hspace{0.25em} =\hspace{0.25em} \frac{1}{{S}_{ij}}\sum_{{\ell}=1}^{L}{IBS}_{ij}^{({\ell})}\hspace{0.17em}{I}_{{\ell}}^{(ij)}$$

where L is the total number of loci, ${I}_{{\ell}}^{(ij)}=1$ if both individuals have non-missing genotypes at locus ℓ and 0 otherwise, and

$${S}_{ij}=\sum_{{\ell}=1}^{L}{I}_{{\ell}}^{(ij)}$$

is the number of valid sites compared between individuals i and j. Finally, the genetic distance is defined as

$${d}_{ij}\hspace{0.25em} =\hspace{0.25em} 1-{IBS}_{ij}.$$

Thus, ${d}_{ij}=0$ indicates identical genotypes across all compared loci, ${d}_{ij}=1$ indicates complete dissimilarity (opposite homozygotes at every locus), and intermediate values reflect partial allele sharing.

## Results

### Cohorts, selection intensity, and phenotype changes

The evaluation method of the lines varied depending on the stage of trialing. Lines in the stage−1 trial were grown in one location with one replication (Table 1). A sparse testing design was implemented for stage−2 trials beginning with the OH17 cohort. Genomic selection was utilized alongside phenotypic selection in the stage−1 and − 2 trials beginning with the OH18 cohort. Routine genotyping of lines in the OSU trials began in 2016 with all lines in the trials in that year. This included only OH13 lines that were in the 2016 stage−3 trials and only OH14 and OH15 lines from the 2016 stage−2 trials. On average, selection intensity was lowest going from stage−1 to stage−2 with 33.5% of lines being advanced and was highest going from stage−3 to stage−4 with 21.3% of lines being advanced (Table 2). Across all stages and cohorts, only 1.9 percent of lines that were in the stage−1 trials were advanced to a stage−4 trial (Table 2).

Significant trait changes during stages of selection were found in most cohorts for grain yield and FHB index, but not for test weight (Table 3). In nine out of ten cohorts, yield increased significantly with an average gain of 143.7 kg/ha per stage of selection. The FHB index decreased by 1.48% per stage of selection.

**Table 3:** Predicted change of trait values as wheat lines from each cohort were advanced from stage−1 to stage−4. The predicted change (*b*) was obtained by regressing the genetic value of each line in a cohort onto number of its most advanced stage of testing (1, 2, 3, or 4)

	Grain yield (kg/ha)		Test weight (g/l)		FHB index (%)	
Cohort	*b*	Sig	*b*	Sig	*b*	Sig
OH13^†^	30.9	*	0.03	ns	2.50	ns
OH14^†^	57.1	***	0.15	ns	− 0.37	**
OH15^†^	190.7	***	0.16	ns	0.32	ns
OH16	261.2	***	0.08	ns	− 4.83	***
OH17	137.7	***	0.45	**	− 2.21	***
OH18	66.5	***	0.84	***	− 0.39	***
OH19	215.6	***	0.03	ns	− 2.08	***
OH20	161.8	***	0.01	ns	− 0.79	***
OH21^†^	57.1	***	0.05	ns		
OH22^†^	122.8	***	0.10	ns		
Average	143.7		0.23		− 1.48	

^†^OH13: Analyses only included lines in stages 3 and 4; OH14 and OH15 only included lines in stages 2, 3, and 4; OH21 only included lines in stages 1, 2 and 3; OH22 only included lines in stages 1 and 2

*,**,*** indicate not significant, significance at probability of 0.05, 0.01, and 0.001 levels

### Markers and population structure

The 1,173 SNPs used in this study were widely distributed across the wheat genome (Table 4). Of the 1173 SNPs, 41% were from the A genome, 44% from the B genome, and 15% from the D genome.

**Table 4:** Summary of marker information by chromosome. # of analyses of allele frequency change (AFC,) % of analyses where AFC was deemed to be due to selection (%AFCsel), % of analyses with AFC >0.05 and < − 0.05), % of markers that showed consistent unidirectional AFC, % analyses where allele frequency became fixed, and the average absolute value of the AFC

Xsome	# Markers	# Analyses*	% AFCsel	% with AFC >0.05	% with AFC <− 0.05	% Consistent	% Fixed	Avg ABS (AFC)
1A	60	2430	25.1	27.4	22.2	3.3	15.3	0.081
1B	77	3117	23.6	24.0	24.3	5.1	14.1	0.075
1D	27	1087	22.1	19.3	19.6	0.0	18.0	0.060
2A	84	3372	23.2	25.4	20.8	3.6	15.2	0.075
2B	77	3108	22.9	23.5	22.0	5.1	14.8	0.071
2D	45	1778	18.5	22.9	13.9	0.0	21.9	0.057
3A	68	2744	22.2	26.2	20.5	1.4	13.6	0.072
3B	102	4130	25.7	28.2	23.2	3.7	13.7	0.083
3D	22	876	26.5	24.0	19.3	0.0	20.1	0.068
4A	50	2032	23.5	23.4	25.3	4.0	10.7	0.075
4B	37	1495	29.2	26.2	26.4	5.2	16.1	0.088
4D	11	442	22.2	15.8	19.2	8.6	16.1	0.058
5A	55	2230	24.1	25.2	22.9	1.6	13.0	0.077
5B	64	2596	25.4	28.1	23.3	4.5	13.5	0.081
5D	12	471	20.4	17.6	14.0	8.5	20.4	0.051
6A	52	2089	24.7	25.4	22.4	1.7	16.5	0.076
6B	81	3255	22.4	26.1	21.8	2.3	16.5	0.073
6D	33	1319	24.3	21.1	19.9	5.5	16.1	0.067
7A	113	4573	24.2	23.5	27.0	2.6	12.6	0.079
7B	73	2945	25.5	24.8	26.2	1.4	12.2	0.082
7D	30	1193	22.0	18.9	20.0	9.8	14.9	0.057

*# of analyses is the number of markers × the number of cohorts/selection scenarios combinations where the marker was assessed

The population structure of the 4,673 lines used in the study was assessed by grouping lines by cohort and testing stage forming 33 groups and using data from 1,173 markers. For example, OH16 lines in stage−2 of testing were placed in the OH16-2 group. The fixation index (F_ST_) was calculated among all 33 groups of lines to assess the degree of genetic differentiation among groups and a cluster analysis was conducted to visualize the association among the 33 groups.

**Fig. 2:**
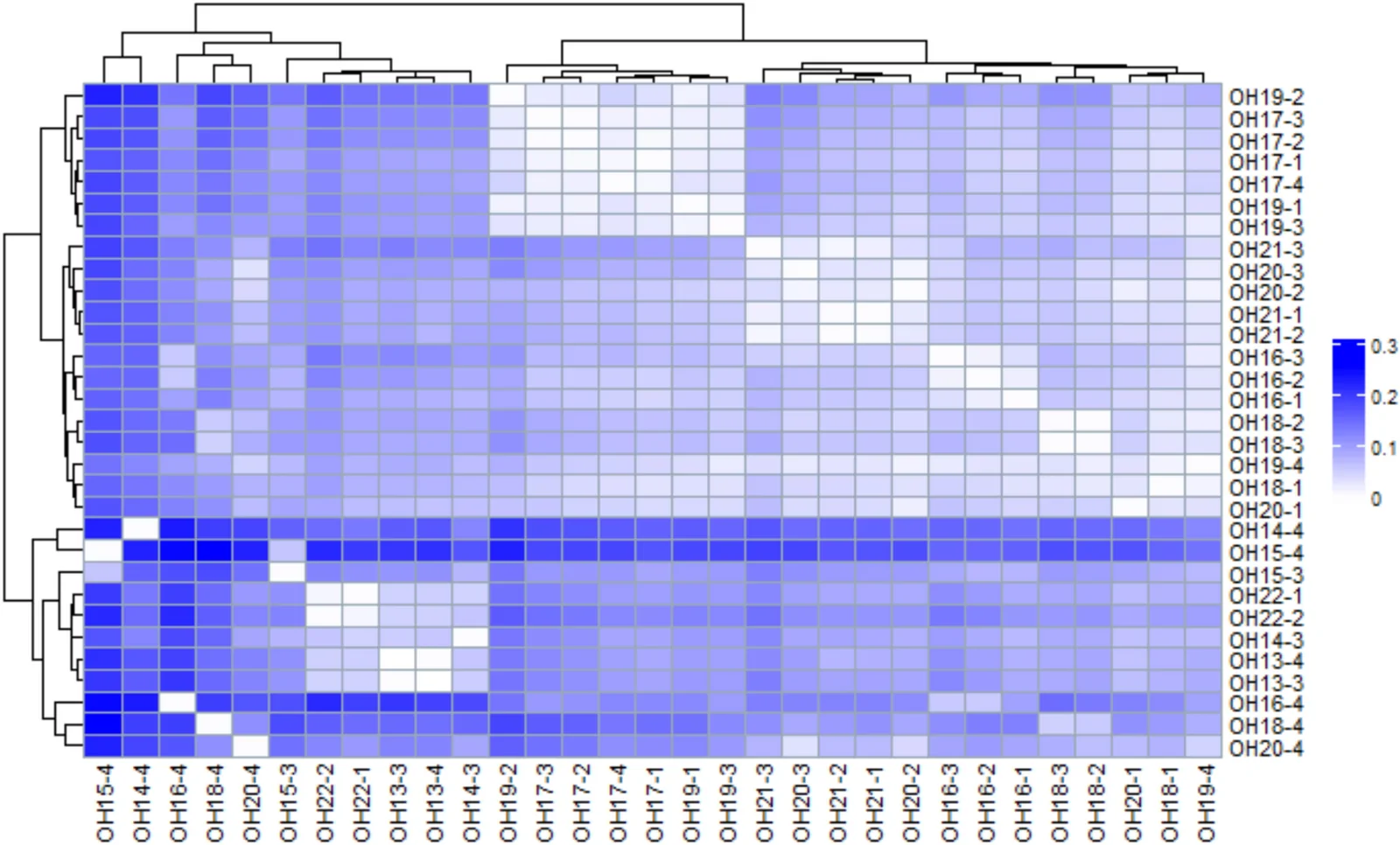
Heatmap of the marker-based genetic distance matrix for 4678 soft red winter wheat breeding lines, grouped by cohort and the last stage of their testing. Identifiers are the cohort code and stage of testing. OH19-2 is comprised of OH19 lines tested in stage−2, OH19-3 is comprised of OH19 lines tested in stage−3

Cluster analyses of the genetic distance matrix displayed two distinct clusters with two subclusters each (Fig. 2). Lines in stage 1, 2, and 3 trials from the same cohort were always in the same sub-cluster. Lines in stage−4 trials only clustered with lines of their cohort from previous stages in the OH17 cohort (with stage−1, − 2, and − 3 lines) and OH13 cohort (with stage−3 and − 4 lines) cohorts.

The average F_ST_ value among the 33 groups was 0.089. The highest F_ST_ value (0.233) was observed between lines in the OH14-4 and OH16-4 sets. The lowest F_ST_ value (− 0.0029) was observed between lines OH13-3 and OH13-4 sets. Within a cohort the average differentiation of stage−1 lines with lines in more advanced stages increased as lines advanced through stages of testing (Table 5). The average F_ST_ between stage−1 and 2 lines was just 0.01, while the average F_ST_ between stage−1 and stage−4 lines was 0.051. This suggests that the stage−4 lines were only moderately differentiated from the stage−1 lines within any one cohort.

**Table 5:** F_ST_ values of wheat lines in one cohort in one stage of testing (stages 1, 2, 3, or 4) versus lines from the same cohort in a different stage of testing

	Stages of comparison					
Cohort	1 vs 2	1 vs 3	1 vs 4	2 vs 3	2 vs 4	3 vs 4
OH13						– 0.001
OH14				0.008	0.041	0.121
OH15				0.010	0.047	0.057
OH16	0.014	0.030	0.094	0.010	0.051	0.051
OH17	0.004	0.009	0.004	0.002	0.014	0.012
OH18	0.021	0.025	0.100	0.001	0.051	0.045
OH19	0.012	0.009	0.033	0.025	0.077	0.017
OH20	0.014	0.034	0.069	0.008	0.035	0.028
OH21	0.002	0.013	0.007	0.008	0.035	0.028
OH22	0.005					
Average	0.010	0.020	0.051	0.009	0.044	0.040

The average F_ST_ among stage−1 lines from different cohorts was 0.05, ranging from 0.014 to 0.105. The average F_ST_ among stage−2 lines was 0.078, ranging from 0.017 to 0.130. The average F_ST_ among stage−3 lines was 0.085, ranging from 0.018 to 0.163. The average F_ST_ among stage−4 lines was 0.154, ranging from 0.043 to 0.260. Selection increased the differentiation among lines from different cohorts with the greatest increase in differentiation (+ 0.069) occurring when advancing from stage−3 to stage−4.

### Allele frequency changes

There were 48,093 marker/cohort/selection stage combinations where we calculated AFC. If the probability of an observed AFC being due to drift was ≤0.05, then the observed AFC was attributed to selection and identified as AFCsel. Of the 48,093 calculated AFC, 22,402 (46.6%) had an AFC whose absolute value was ≥ 0.05 and 11,301 instances (23.5%) had an AFC that were deemed to be AFCsel: Such instances were distributed across the genome (Table 4).

Each marker had a maximum of 41 scenarios (cohort/stage of selection combination) where the observed AFC could be compared to that expected by drift. A marker was deemed to show consistent signs of unidirectional selection across cohorts and stages of selection as described in the materials and methods. For example, for marker S2A_32554951, 73.2% of its 41 AFCs were classified as AFCsel and an AFC of >0.05 occurred 30 times more often than an AFC of <− 0.05: The tracked allele at S2A_32554951 was consistently being favored during line advancement and apparently due to selection. Based on these two criteria, we determined that 91 of the 1,173 markers showed a consistent signature of directional selection (Supplementary Table 1). The average linkage disequilibrium ${r}^{2}$ value among the 91 markers declared to show consistent signature of selection was 0.052. Only two pairs of markers had an ${r}^{2}$ value that was greater than 0.4: One marker from each of the two pairs was removed from the list of consistent markers leaving 89 apparently independent markers with consistent AFCs (Supplementary Table 1). Of the 11,301 AFC determined to be attributed to selection, 3,584 (31.7%) were from the 89 consistent markers, meaning that 68.3% of the AFCsel were specific to a particular cohort and selection stage scenario.

We performed an analysis of variance (ANOVA) to assess the impact of cohort and stage of testing on several metrics of AFC. The stage of selection had a significant (*p* < 0.05) effect on %AFCsel, number of markers with AFC values of 0.05 or − 0.05, percentage of markers that became fixed, and absolute value of AFC (Table 6). Cohort only had a significant effect on the absolute value of AFC. Most notably, any selection involving advancing line to stage−4 (ie., 1→4, 2→4, 3→4) had a large impact on the metrics with advancing lines from stage−3 to stage−4 likely accounting for most of the 1→4 and 2→4 effects (Table 7).

**Table 6:** Analysis of variance of the effect of selection scenario and cohort on the parameters used to assess allele frequency changes over all markers

Parameter	F-Test of factor	
	Stage of selection	Cohort
% markers with Prob. of drift <0.05	1.8 ns	0.97 ns
% markers with AFC >0.05	27.1***	1.48 ns
% markers with AFC <− 0.05	16.0***	1.05 ns
% markers fixed	27.6***	1.00 ns
Absolute value of marker AFC	1724***	286***

ns, and *** indicate non-significance and significance at *p* < 0.001, respectively

**Table 7:** Average values of parameters used to assess allele frequency change (AFC) by stage of selection including number of observation, percentage of instances where AFC was deemed to be due to selection (%AFCsel), percent of instances with AFC <0.05 or <− 0.05, percentage of instances where an allele was fixed, and the average absolute value of the AFC (ABS(AFC). Values within a column with the same letter are not significantly different based on a Duncan’s multiple range test with* P* <0.05

Advance-ment	# Obser-vations	% AFCsel	% with AFC > 0.05	% with AFC < − 0.05	% Fixed	Avg. ABS (AFC)
1→2*	8199	36.9b	9.6a	10.9c	2.4 b	0.03a
1→3	7029	29.0ab	21.9b	21.9b	5.8 b	0.06b
1→4	5859	21.1ab	38.0c	30.6a	27.1 a	0.12c
2→3	9286	19.9ab	14.3ab	16.7b	4.5 b	0.04d
2→4	8148	20.4ab	35.9c	29.5a	27.5 a	0.12e
3→4	8761	17.0a	33.1c	28.6a	24.1 a	0.10f

*1→2, indicates advancing lines from stage−1 trials to stage−2 trials; 1→3 indicates advancing lines from stage−1 trials to stage−3 trials, etc.

Values within each row are summations over all markers and cohorts where such selection occurred

A GWAS was conducted using BLUEs of all lines from all cohorts for grain yield, test weight, and FHB index. Markers were deemed “significant” if their probability from the GWAS was < 0.05. A Chi-square test was used to compare the observed distribution of significant markers among the 89 markers with consistent AFC and the distribution expected from the 1084 “inconsistent” markers. The χ^*2*^ tests showed that the frequency of significance among the 89 consistent markers was greater than expected yield, FHB index and test weight at *p* < 0.05.

### Fixation and genetic distance

**Fig. 3:**
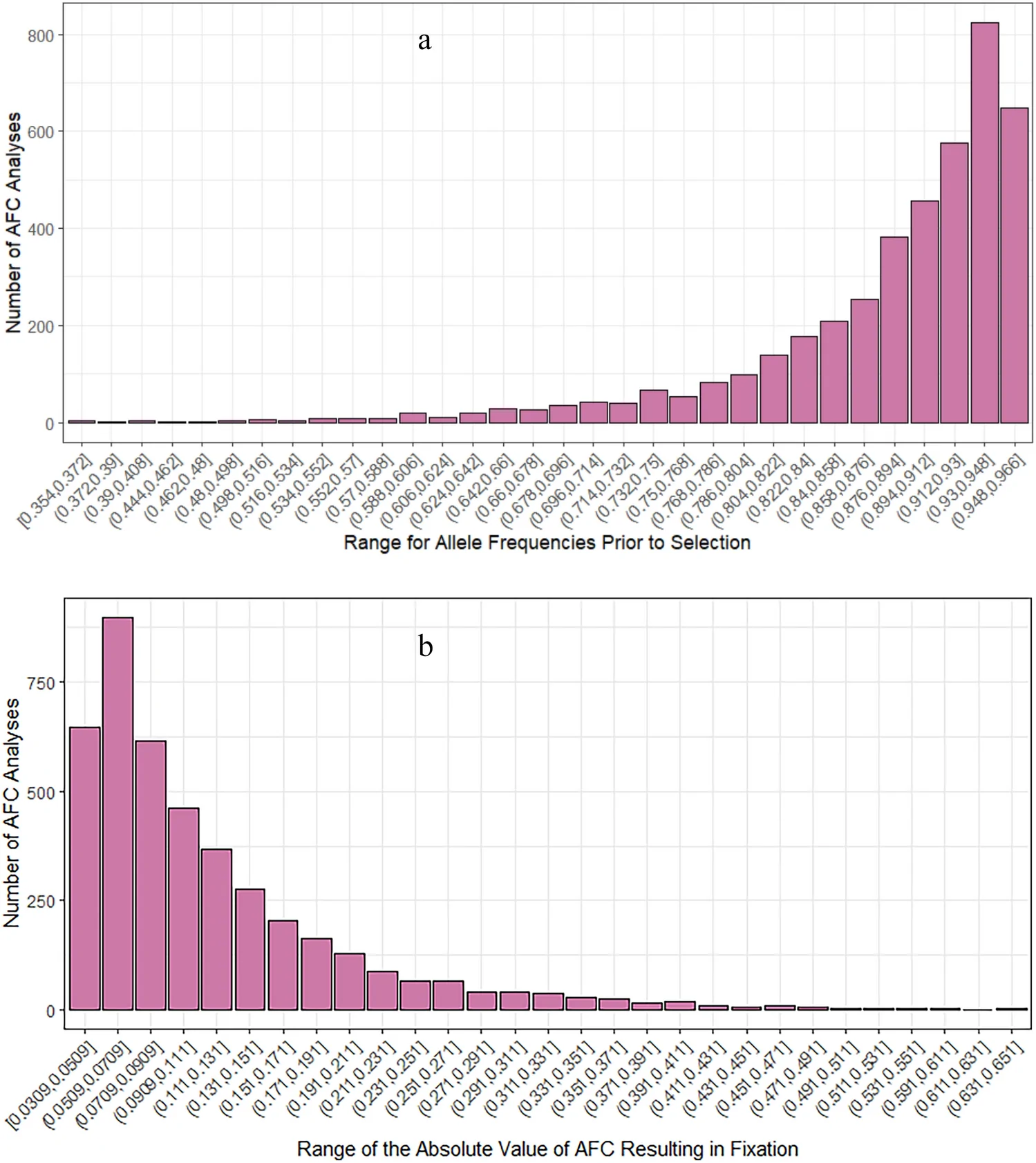
a and b Distribution of initial allele frequency **a** and absolute value of allele frequency changes (AFC) **b** from all marker/cohort/selection stage scenarios that resulted in allele fixation

A marker was declared fixed when its initial MAF was < 0.96 and its final MAF was > 0.99. Fixation was assessed for all markers within each cohort and each stage of selection. The percentage of markers that became fixed varied by chromosome (Table 4) and was significantly affected by stage of selection, but not by cohort (Table 6). An average of 14.7% of all markers became fixed within each cohort, ranging from 3.28% in OH22 to 25.37% in OH14 (Table 7): note, the OH22 cohort only went through one stage of selection (1→2). On average, the initial allele frequency of a marker in the testing stage prior to fixation was 0.883 (median 0.911, range 0.3.544 to 0.9599) (Fig. 3A). The average absolute value of the AFC that resulted in fixation was 0.1166 (median 0.089, range 0.03093–0.6456) (Fig. 3B).

The percentage of markers that became fixed increased as lines were advanced from stage−1 to stage−4. Over all cohorts and lines, just 11% of all markers were fixed in stage−1, 15.3% in stage−2, 24.57% in stage−3, and 28.7% in stage−4. The percentage of markers fixed was greatest in all selection scenarios that involved advancement to stage−4 (i.e., 1→4, 2→4, and 3→4) (Table 7). The stage−3→4 advancements alone resulted in fixation of an average of 24.1% of all marker/cohort combinations (Table 7). Of all AFCs that resulted in fixation, 65.5% occurred when advancing lines from stage−3 to stage−4. We analyzed the 3→4 advancements in eight cohorts (Table 1) and assessed whether a marker that became fixed in one cohort was likely to become fixed in another cohort. A total of 174 markers in these eight cohorts were fixed during the 3→4 advancement stage, and on average, 25.1% of the 174 markers that became fixed in one cohort also became fixed in another cohort. Over all cohorts there were 84 lines that entered the stage−4 trials and just 1.97% of all markers were fixed over these 84 lines.

The high rate of allele fixation within a cohort suggested a severe loss of diversity during selection. When combined over all cohorts, the genetic distance between lines declined over stages of testing from an average of 0.291 among all stage−1 lines to 0.275 among all stage−4 lines, a 5.5% decline (Table 8). We calculated the genetic distance between lines from different cohorts at each stage of testing. The average genetic distance between stage−1 lines from different cohorts was 0.293 and among stage−4 lines from different cohorts it was 0.290. The distance was greatest among stage−3 lines from different cohorts (0.296).

**Table 8:** The average genetic distance between lines from different stages of testing and from different cohorts

Cohort		Genetic distance				
		Stage−1	Stage−2	Stage−3	Stage−4	Average
OH16	OH17	0.294	0.289	0.289	0.285	0.289
OH16	OH18	0.294	0.291	0.293	0.286	0.291
OH16	OH19	0.295	0.294	0.292	0.286	0.291
OH16	OH20	0.298	0.295	0.285	0.294	0.296
OH17	OH18	0.293	0.296	0.295	0.293	0.294
OH17	OH19	0.283	0.271	0.277	0.296	0.283
OH17	OH20	0.296	0.297	0.303	0.300	0.299
OH18	OH19	0.291	0.300	0.291	0.284	0.300
OH18	OH20	0.295	0.290	0.290	0.276	0.290
OH19	OH20	0.293	0.295	0.295	0.287	0.292
Average		0.293	0.292	0.296	0.290	
Average GD over all lines from all cohorts		0.291	0.287	0.284	0.275	0.284

For example, the genetic distance between the stage−1 lines from the OH16 cohort and stage−1 lines from the OH17 cohorts was 0.294. The last row presents the average genetic distance among all lines from all cohorts that were tested in each stage

## Discussion

Advancing (selecting) lines through the stages of field trials produced significant improvement for yield and FHB index in nearly every cohort (Table 3), but little change in test weight. This is likely because the program aims to improve yield and FHB resistance while being content to maintain test weight at its current level. The selection was primarily based on phenotypes, especially as lines advanced from stage−2 to stage−3 and from stage−3 to stage−4.

Our primary objective was to assess the impact on AFC from advancing lines through stages of trialing. This was done by tracking AFC of 1,173 markers in ten cohorts comprised of 4,365 lines and six selection scenarios. It was clear that advancing lines through stages of testing and the effective phenotype improvement resulted in large genome changes. Many changes were documented with 23.49% of analyses showing an AFC greater than expected by drift. The large changes we document occurred after just three stages of phenotypic selection. In maize, Wang et al. (2020) reported that 50% of assayed loci were affected by selection for multiple agronomic traits. Moose et al. (2004) reported that 20% of loci showed signs of selection over 100 cycles of selection in the Illinois long-term maize oil and protein selection population. Others have also noted the widespread impact of selection on the genome (Innan and Kim 2004; Johansson et al. 2010).

We found 89 markers (7.59%) that showed consistent signs of unidirectional selection across cohorts, stages of selection, years and environments (Supplementary Table A). Our GWAS analysis indicated that these 89 markers were more likely to be associated with the selected traits than were markers with “inconsistent” AFC, though no markers had a large effect on any trait. It is possible that the 89 markers simply have small, but consistent effect on yield and resistance to FHB, and thus are not declared significant in the GWAS. Further research is necessary to discern the impact of these consistent markers on traits and how this information can be used in selection. The consistency of the AFC of the 89 markers is noteworthy and these markers account for just 31.3% of the 11,301 AFC attributed to selection, while the other and 68.7% were specific to particular marker/cohort/selection-stage scenarios. This suggests a prevalence of marker x environment interaction, and epistatic effects on AFCs.

Our percentage of markers with consistent AFC aligns with the findings from Mondaini et al. (2022), where 9.8% of loci in wheat displayed strong evidence of selection over time, and 2% of loci displayed strong evidence of selection over environments and trials over 38 years of the CIMMYT bread wheat breeding program. N’Diaye et al. (2018) analyzed loci under selection in a durum wheat (*Triticum turgidum subsp. durum*) breeding program and also identified a similar percentage of SNPs (9.6%) that appeared to be affected by selection. Our study differs from those of Mondaini et al. (2022) and N’Diaye et al. (2018) as we assess AFC over stages of selection with cohorts instead of only elite lines derived from decades of breeding. Our results show that many additional loci are affected by selection in a situation-specific fashion during advancement of lines through stages of testing.

The greatest impact of selection on the genome occurred when advancing lines from stage−3 to stage−4 (Table 7). The number of loci that became fixed and the magnitude of the AFC was greatest when advancing lines from stage−3 to stage−4 than in any other advancements (Table 7). This was also manifested in the population structure. The average F_ST_ among stage−1, 2 and 3 lines within a cohort were less than 0.021, which indicates low differentiation (Table 5) (Frankham et al. 2002), and stage−1, 2 and 3 lines from the same cohort clustered together (Fig. 2). The average F_ST_ involving stage−4 lines were all greater than 0.039 (Table 5) and stage−4 lines within a cohort rarely clustered with the other members of the cohort.

Allele fixation within a cohort occurred most often when advancing lines from stage−3 to stage−4, which suggests that the use of phenotypic selection during the later stages trialing phase had a significant impact on diversity. Selection intensity in the OSU wheat breeding program was greatest when advancing lines from stage−3 to 4 (21.3%) though it was not greatly different advancing lines from stage−2 to 3 (23.6%) (Table 2). It is also possible that the high fixation rate in the stage−3 to stage−4 selection is due to the use of high heritability, multi-years phenotypic data in selection. Such data are more indicative of genetic value than phenotypes from earlier stage trials so the selection is more effective and would cause larger AFC.

The degree of allele fixation within a cohort that occurs by stage−4, the accompanying large AFC values, (Table 5), and general decline of diversity (last row, Table 8), could have a significant negative impact on long term genetic gains. The negative impact could be mitigated by selecting parent lines earlier in the breeding pipeline where fixation rates are low. A drawback with selection in early stages is that the phenotypic selection would be based on less phenotypic data and lower heritability than selection at later stages and this could reduce gain per cycle, though perhaps improve gain per year. Alternatively, one could use novel parent selection approaches such as genomic optimal contribution (Meuwissen et al. 2020).

The loss of diversity by stage−4 would be a significant issue in true recurrent selection involving closed populations, as all parents for the next cycle selected from the same cohort. But few breeders practice true recurrent selection. In the OSU wheat breeding program, we select parents to initiate new crosses from several recent cohorts. We also cross with parents from other programs. For example, the lines in the OH22 cohort are not derived from the best lines in the one cohort. About 85% of the parentage of OH22 lines comes from OH12, OH13, and OH14 cohorts and 15% come from non-OSU, albeit related, sources. The best lines are used as parents for several successive cohorts, a practice that creates connectivity among the cohorts and enhances the accuracy of genomic selection. Thus, each cohort has a unique, though also somewhat overlapping, set of founders. While fixation was high within a cohort, few alleles became fixed in multiple cohorts, and the genetic distance among stage−4 lines was not much lower than among all stage−2 lines.

The average F_ST_ between stage−4 lines from different cohorts was 0.154, which would be considered highly differentiated (Frankham et al. 2002). As the stage−1 lines from different cohorts are not very differentiated (average Fst = 0.049), then the observed increased divergence among stage−4 lines from different cohorts likely arises from being tested and selected in different sets of locations and years. For example, selection of OH17 lines from stage−1, 2 and 3 trials occurred in 2018, 2019, and 2020 while the selection of OH18 lines from stage−1, 2, and 3 trials occurred in 2019, 2020, and 2021: The OH17 and OH18 selections would share just four of 14 trailing environments (year/location combinations). These spatial and temporal differences among selection environments, primarily for yield, may cause enough difference among environmental and genotype × environment interaction (G×E) effects to cause genetic differentiation among the stage−4 lines. The diversity among stage−4 lines from different cohorts appears to cause stage−1 lines from different cohorts to be fairly undifferentiated from one another, indicating that there is gene flow between adjacent cohorts, as parents and their subsequent offspring are reused in overlapping cycles of crossing (Suzuki 2010). Crossing between the best lines from different cohorts appears to restore nearly all the diversity lost within one cohort and reintroduces allelic variation to loci that become fixed within a single cohort. It also recombines alleles that have been selected over a wide array of environments and genetic backgrounds which may enhance developing unique cultivars. It is common for breeders to cross among the best lines from different cohorts. Our results indicate that this practice can quickly restore diversity lost during advancement. The practice will also recombine alleles with consistent directional AFC across a broad sample of environments as well as alleles where AFC were environment specific.

## Conclusions

Plant breeders often grapple with the dilemma of balancing selecting elite lines to attain immediate goals, and maintaining, or even increasing, genetic diversity to sustain long-term progress. Phenotypic selection within a breeding pipeline led to significant changes to the wheat genome with the greatest changes occurring when selecting the best lines from stage−3 to enter stage−4 testing. We identified 89 markers that showed consistent signs of unidirectional selection over cohorts, stages of selection, and environments. The value of these loci in genomic breeding needs to be investigated in subsequent studies. While there was considerable allele fixation within a cohort, fixation was limited when assessed across stage−4 lines from all cohorts. The selection caused considerable differentiation among stage−4 from different cohorts and fixation of alleles within cohorts. Allele fixation though was limited when assessed over stage−4 lines from all cohorts and there was considerable diversity among these lines. The diversity likle arises from selection in varying genetic backgrounds and environments. Crossing among the best stage−4 lines from multiple cohorts can be used to exploit their differentiation and create ample genetic diversity for the next cycles of selection.

## Supplementary Information

Below is the link to the electronic supplementary material.

Supplementary file1 (XLSX 23 kb)

## Data Availability

All data are available from Dr. Clay Sneller, sneller.5@osu.edu.
